# Improving diagnosis-based cost groups in the Dutch risk equalization model: the effects of a new clustering method and allowing for multimorbidity

**DOI:** 10.1007/s10754-023-09345-0

**Published:** 2023-03-02

**Authors:** Michel Oskam, Richard C. van Kleef, René C. J. A. van Vliet

**Affiliations:** Erasmus School of Health Policy & Management, Erasmus University Rotterdam, Rotterdam, The Netherlands

**Keywords:** Health insurance, Risk equalization, Risk selection, Risk adjustment, I10-health, G22-insurance, Insurance companies, Actuarial studies, H51-Government expenditures and health

## Abstract

Health insurance markets with community-rated premiums typically use risk equalization (RE) to compensate insurers for predictable profits on people in good health and predictable losses on those with a chronic disease. Over the past decades RE models have evolved from simple demographic models to sophisticated health-based models. Despite the improvements, however, non-trivial predictable profits and losses remain. This study examines to what extent the Dutch RE model can be further improved by redesigning one key morbidity adjuster: the Diagnosis-based Cost Groups (DCGs). This redesign includes (1) revision of the underlying hospital diagnoses and treatments (‘dxgroups’), (2) application of a new clustering procedure, and (3) allowing multi-qualification. We combine data on spending, risk characteristics and hospital claims for all individuals with basic health insurance in the Netherlands in 2017 (N = 17 m) with morbidity data from general practitioners (GPs) for a subsample (N = 1.3 m). We first simulate a baseline RE model (i.e., the RE model of 2020) and then modify three important features of the DCGs. In a second step, we evaluate the effect of the modifications in terms of predictable profits and losses for subgroups of consumers that are potentially vulnerable to risk selection. While less prominent results are found for subgroups derived from the GP data, our results demonstrate substantial reductions in predictable profits and losses at the level of dxgroups and for individuals with multiple dxgroups. An important takeaway from our paper is that smart design of morbidity adjusters in RE can help mitigate selection incentives.

## Introduction

Many social health insurance systems rely on regulated competition to enhance fairness and efficiency in health care financing (Ash et al., 1989; Enthoven, 1988). Two typical regulatory features include premium-rate restrictions and risk equalization (RE). Rate restrictions—e.g. in the form of community-rating per insurance plan—protect individual affordability of health insurance for high-risk people who would otherwise be charged (very) high premiums. RE compensates insurers for the predictable variation in individual health care spending using a predefined set of risk adjusters such as the age, gender and health status of enrollees. Appropriate compensation is required to level the playing field for competing insurers, reduce the selection incentives they face and protect the functioning of the insurance market (Rothschild & Stiglitz, 1976; Newhouse, 1996; Van de Ven & Ellis, 2000; Layton et al., 2018c).

Although RE systems have developed from simple demographic models (adjusting only for age and gender) to sophisticated morbidity-based models—containing indicators based on inpatient diagnoses, pharmaceutical usage and prior care spending –, recent studies have demonstrated that even advanced systems do not eliminate selection incentives (Van Kleef et al., 2018; McGuire et al., 2020). For example, the most sophisticated RE models, applied under the Affordable Care Act in the United States (U.S.) and in the basic health insurance schemes in Germany and the Netherlands, undercompensate insurers for specific, sizable groups of chronically-ill consumers such as diabetics and patients with heart diseases (McGuire et al., 2020; Withagen-Koster et al., 2020).

Therefore, the aim of this paper is to diminish the persisting under/overcompensations of the Dutch RE model through the redesign of one of its key risk adjusters for morbidity, the Diagnosis-based Cost Groups (DCGs). This morbidity adjuster classifies individuals in cost groups based on inpatient and outpatient diagnoses and treatments. All selected codes relate to expected medical spending in the subsequent year and are grouped into 200 clusters of diagnoses called ‘*dxgroup*s’, based on clinical homogeneity. These dxgroups, in turn, are clustered into the aforementioned DCGs based on their respective residual spending from a prediction model for health spending that includes age, gender, and an adjuster for pharmaceutical usage (Lamers, 1998; Prinsze & Van Vliet, 2007). These DCGs are then used in the Dutch RE model that includes all risk adjusters. Although this approach is extensive, there is scope for improvement.

A potential drawback of the current DCGs is that the clustering of multiple dxgroups into one DCG inherently subjects the formed DCG to internal heterogeneity, resulting in compromised precision of compensation for specific, included diagnoses. For instance, Van Kleef et al. (2020a) found leukemia patients to be substantially undercompensated (€3,733 on average per person per year), despite inclusion of the diagnosis ‘leukemia’ in one of the DCGs. A second potential drawback of the current DCGs is the limited ability for financial compensation of multimorbidity. Contrary to diagnosis-based risk adjusters applied in American and German RE models in which enrollees can qualify for multiple diagnostic groups (referred to as Hierarchical Conditions Categories and Hierarchical Morbidity Groups respectively), Dutch enrollees are bound to a two-layered classification system of Primary and Secondary DCGs (pDCG/sDCG) where individuals can be flagged by only one DCG per layer (Van Kleef et al., 2018). As a result of this restriction, individuals with multimorbidity are on average undercompensated (Eijkenaar et al., 2018). Moreover, the heterogeneity within the clusters results in imperfect compensation on the level of individual dxgroups. With dxgroups derived from diagnoses and treatments, insurers have the potential to exploit knowledge on (un)profitability of specific treatments when contracting care services. Through selective contracting, insurers could attempt to deter undesirable consumers by abstaining from contracting optimal-performing care providers for specific, unprofitable treatment groups (Layton et al., 2018b). This service-level selection undermines the functioning of the insurance market (Layton et al., 2018a).

The contribution of this paper lies in a new method for clustering the dxgroups to address the issues discussed (i.e., the heterogeneity within DCGs and the lack of multi-classification, and the selection problems that result). The essence of our approach is that we derive more accurate payment weights per dxgroup and use these for a more precise clustering into DCGs. Moreover, we allow for multi-classification: individuals appearing in a total of *n* dxgroups will qualify for *n* DCG flags and enable the corresponding insurer-compensation. The expectation is that these modifications result in better compensations for chronically ill individuals, both on the level of dxgroups and on the level of disease groups. The methods we apply consist of four steps: deriving the baseline RE model as applied in the Netherlands for the year 2020, revising the dxgroups that are included in assembling the DCGs to update the 2020 model, developing and testing the updated clustering method, and finally, evaluating the effects of the new method.

As another novelty, following recent advances in evaluation metrics, this study goes beyond analyzing the effect of improving the DCGs on traditional metrics such as the R-squared (R^2^) and Cumming’s Prediction Measure (CPM). In addition to these metrics, this paper examines the effect on the under/overcompensation of groups that are potentially vulnerable to risk selection, given the characteristics of the Dutch health insurance system. These include the dxgroups underlying the DCGs as well as a broader set of disease groups identified through patient records from Dutch general practitioners (GPs). Under/overcompensation on the level of dxgroups indicate incentives for service-level selection (e.g., not contracting the best doctors for specific treatments) while under/overcompensation on the level of disease groups more generally indicate incentives for group-level selection (e.g., selective marketing and group discounts for supplementary insurance) (Layton et al., 2018a). By examining the effects of our DCG update on both types of selection incentives, a broader insight of its potential strength is acquired. Clearly, the findings of our study are directly relevant for the Netherlands. Additionally, the international relevance of our analyses is to be found in the general conclusion that smart design of morbidity adjusters can help mitigate incentives for risk selection.

The remainder of this paper is organized as follows: first, background context of the history and current (2020) practice of the Dutch DCGs is provided to describe the reasoning for our study. Next, the data and methods are explained, followed by the outcomes of our analyses. The paper closes with a discussion of the conclusions and the resulting implications.

## The Dutch DCGs: history and current practice

### History

Since their initial implementation in 2004, the Dutch DCGs have seen multiple revisions but have always retained the clustered composition. The original 13 DCGs from 2004 contained 69 dxgroups, derived from a selection of ICD-coded diagnoses from inpatient hospital treatments (Prinsze & Van Vliet, 2007). However, adaptations to the funding structure of hospital treatments in 2005 changed the ICD-coding to the so-called ‘diagnosis treatment combinations’ (DTC), which called for a revision of the DCGs and the underlying dxgroups. Moreover, further changes to the DCGs were made in 2009 and 2012 to adhere to changes in the DTCs, seeking to capture shifting patterns in health care utilization (Van Kleef et al., 2014). The update in 2012 led to discarding of the exclusivity of *inpatient* hospital diagnoses. By extending the DCGs to diagnoses from *outpatient* treatments by medical specialists, the predictive accuracy of the RE model increased and the undercompensations for the chronically ill declined. The expansion of DCGs resulted in a surge in prevalence: 2.7% of consumers qualified for compensation through the inpatient DCGs, while 8.5% qualified after the modification.

In a more recent update in 2015, the number of dxgroups that make up the DCGs—which had incrementally risen from 69 to 143—was increased to 189 in order to cover for expansions of the benefit package and new changes in the coding system (Van Vliet et al., 2015). As a result of the increase, 9.4% of the population now qualified for DCG compensation. The most recent update of the DCG classification was performed in 2018 when the split was made between primary and secondary DCGs in an effort to compensate for comorbidity through a new maximum of two DCG flags per enrollee (before, only one was counted, i.e. the one with the highest mean residual spending). In addition, the number of dxgroups further increased from 189 to 200 (Eijkenaaret et al., 2018). With the adjuster now including 15 pDCGs and 7 sDCGs, 10.5% of the population qualifies for at least one of the DCGs.

### Current practice

Since the DCGs are designed to predict health spending in the subsequent year through disease classification, the technique to derive an accurate estimation for the adjuster is both a clinical and statistical endeavor (Ash et al., 1989; Van Kleef et al., 2018). First, all relevant information on hospital care is gathered to separate the primary, unambiguous diagnoses, by excluding stomach complaints, for instance, and including diabetes. Second, a medical judgment is made by experts to select diagnoses that reflect a chronic condition and recurring health spending. The resulting diagnoses are then clustered to DCGs in order to minimize complexity of the RE model and simultaneously mitigate incentives for gaming (Prinsze & Van Vliet, 2007). Medical experts cluster the diagnoses into clinically more or less homogeneous dxgroups, which are subsequently clustered into DCGs by their respective residual spending from an Ordinary Least Squares regression for health spending. The clustering is performed with Ward’s hierarchical clustering method which continuously merges two clusters whose fusion results in a minimal increase in variance in mean residual spending within the newly found cluster (Lamers, 1998). This way the heterogeneity within the new clusters (DCGs) is minimized. Thus, while the clinical funnel-approach serves to select medically valid, unambiguous diagnoses that relate to future spending, the statistical counterpart is applied to derive clusters of dxgroups that maximize model fit (Ellis & Ash, 1995).

Despite the careful selection of diagnoses and the statistical procedure of clustering, undercompensations for groups of chronically ill remain. The work by Eijkenaar et al. (2018) partly addressed the heterogeneity within the DCG classification by considering comorbidity through a maximum of two DCGs (one pDCG and one sDCG) per person per year. In an effort to further improve the DCG adjuster, the authors ventured beyond the two-layered approach by deriving a model including all 200 dxgroups as separate risk adjusters (in the form of dummy variables indicating whether or not an enrollee is flagged by a dxgroup). Although this strategy improved model fit and de facto eliminated heterogeneity among dxgroups clustered in the same DCG, it was considered too complex from a practical point of view given the number of variables and the instability of payment weights for uncommon dxgroups. Therefore, we seek to reduce heterogeneity within the DCG-classification without aggravating concerns related to complexity and instability.

## Data

For this study, three datasets have been made available which can be merged through an anonymized identification key. The first dataset includes information on spending and risk adjusters for all roughly seventeen million individuals with basic health insurance in the Netherlands in 2017. This dataset has been used for the official calibration of the Dutch RE model for 2020. The second dataset covers the roughly twenty million individual hospital claims for specific hospital diagnoses in 2016, of which around five million are relevant for the DCG-classification of 2020. The third and final dataset includes morbidity information from electronic patient records of about 400 Dutch GP practices. In total these practices serve 1.3 million individuals. For each of these individuals the dataset includes morbidity information according to the International Classification of Primary Care (ICPC) (WHO, 2021). Specifically, the dataset indicates whether or not an individual suffered from a specific chronic illness in 2016. This dataset will be used to identify disease groups for which we will calculate under/overcompensations using the alternative models. It is noteworthy that the patient records by the GP provide a valuable insight into the health status of Dutch individuals. In the Netherlands, the GP serves a gatekeeping role towards specialized (hospital) care and prescribes the use of outpatient drugs.

Since the GP dataset is essentially a subsample of the calibration dataset (N = 1.3 m versus N = 17 m), the GP subsample has been rebalanced to reflect the calibration dataset. Through a procedure of data reconciliation, the target dataset (GP data) is made to reflect the initial dataset (Dutch population) in terms of a predefined set of variables (Battagliaet et al., 2009). The iterative fitting procedure determines a weighting factor for every observation within the target dataset based on the risk classes of the equalization model (discussed in “Methods” section) After applying the weighting per individual in subsequent calculations, the target dataset reflects frequencies of risk classifications and spending that are consistent with the total population of seventeen million (for a more detailed description of the procedure, see Van Kleef et al., 2020a). Moreover, the variables unique to the target dataset have their frequencies enlarged to an amount that reflects the complete population, based on the weighting factors.

## Methods

To modify the DCGs and to examine the effects of the modified DCGs, the methodological buildup of this study consists of four steps: (1) derive a baseline model (i.e. the actual Dutch RE model for 2020), (2) revise the dxgroups underlying the DCGs and evaluate the effect thereof on measures of explanatory power, (3) develop a new clustering method for deriving DCGs and evaluate its impact on explanatory power, and (4) evaluate the effects of the adjustments to DCGs in terms of the under/overcompensations for subgroups that are potentially vulnerable to risk selection. In the following part the respective steps are explained in greater detail, describing the different models used in this study.

### Step 1 Deriving a baseline model: the actual Dutch RE model of 2020

In order to set up a valuable comparison between the status quo and the proposed modifications to the model, we first calibrate a baseline model. This model (which we refer to as Model 0, or M0) mimics the official Dutch RE model of 2020. For simplicity, we solely focus on the RE model for somatic care, accounting for roughly 90 percent of spending in basic health insurance.^1^ This model includes eleven risk adjuster classifications with nearly 200 risk classes in total (Van Kleef et al., 2018; Staatscourant, 2020). Based on the coefficients that result from an individual-level regression of health spending on these risk classes, a prediction of health spending in 2020 is made.

The risk adjusters in the baseline model include age interacted with gender, pharmacy-based cost groups (PCGs), DCGs, durable medical equipment groups, institutional status interacted with age, clusters of zip-codes based on regional factors, socioeconomic status interacted with age, household size interacted with age, multiple-year high cost groups, physiotherapy diagnosis groups, and prior spending on home care (Van Kleef et al., 2018). Of these classifications, most are either based on demographic information or derived from information on hospital utilization. However, the role of primary care is implicitly considered in the model as well, since the PCGs are derived from drugs prescribed by GPs. So, through these PCGs the RE model compensates for the above-average spending of chronically ill people who were not treated in a hospital in the prior year but by their GP, e.g. (most) diabetics.

### Step 2 Revision of dxgroups underlying DCGs

After deriving the baseline model, the next steps cover the adaptations to the model. In line with the historically recurring revisions to the DCGs, the first change to the model was to update the dxgroups on both clinical and statistical grounds. The revision incorporates similar criteria as used by Ellis and Ash (1995): at least a third of the patients with the considered diagnoses need to be diagnosed in two consecutive years, the diagnoses need to be unambiguous and the average related health spending and prevalence both need to exceed predefined thresholds. The resulting selection of diagnoses was then evaluated by a committee of clinical experts, leading to a clustering of the diagnoses to clinically meaningful dxgroups. This revision process increased the number of dxgroups from 200 to 209—removing 10 and adding 19-, mostly because of heterogeneity in the expenditures for diagnoses in the same clustered dxgroup that therefore needed to be separated (for the full report—in Dutch—that delves deeper into the selection of dxgroups, see: Van Kleef et al., 2020b).

To analyze the effects of the revision, we estimated an updated version of the baseline model. In this Model 1 (or M1), the DCGs of 2020 are replaced by the updated DCGs from step 2, built with the new total of 209 dxgroups. This method follows the exact same route to compile DCGs as the baseline model, as discussed in the Introduction and “Current practice” section. Therefore, M1 has the same number of primary (15) and secondary (7) DCGs as M0 but will inevitably have different respective coefficients. We compare the explanatory power of this updated model with that of the baseline model using R^2^ and CPM, which are calculated according to (1) and (2) respectively.

1 $${\text{R}}^{2} = 1 \, - \frac{{\mathop \sum \nolimits_{i = 1}^{n} \left( {y_{i} - \hat{y}_{i} } \right)^{2} }}{{\mathop \sum \nolimits_{i = 1}^{n} \left( {y_{i} - \overline{y}} \right)^{2} }}$$

2 $${\text{CPM}} = 1 \, - \frac{{\mathop \sum \nolimits_{i = 1}^{n} \left| {y_{i} - \hat{y}_{i} } \right|}}{{\mathop \sum \nolimits_{i = 1}^{n} \left| {y_{i} - \overline{y}} \right|}}$$

In both equations, $y_{i}$ represents the actual spending for individual *i*, $\hat{y}_{i}$ the predicted value of spending for *i* based on the respective RE models (M0, M1, etc.) and $\overline{y}$ the average spending in the population (Layton et al., 2018a). For both measures, a value closer to 1 indicates higher explanatory power of the model. The difference between (1) and (2) is that CPM handles errors in the regression model linearly whereas R^2^ gives a larger weight to larger errors.

### Step 3 Developing and testing a new method of clustering dxgroups into DCGs

The third step of our empirical analysis concerns the design and evaluation of a new approach for clustering dxgroups into DCGs to mitigate the earlier described problems of heterogeneity. The new method of clustering is comprised of two phases. First, all 209 dxgroups derived from step 2 are included as separate risk adjusters in the process of calibrating the actual RE model. To be precise, the 209 dxgroups serve as explanatory variables, like age, gender and PCGs, for the individual-level regression of health spending to obtain *coefficients*. By doing so, the respective coefficients for dxgroups are corrected for all other adjusters. In contrast, the traditional method (as used for M0 and M1, discussed in step (2) uses a partial model that corrects for age, gender and PCGs alone, to obtain *mean residual spending* for each separate dxgroup and clusters these to create the DCGs for the complete model with all adjusters. Through the new method, the respective coefficients are corrected for multimorbidity as well as potentially confounding effects of the other risk adjusters.

In the second phase of the new method, the 209 dxgroups are clustered into 26 DCGs on the basis of their estimated coefficients. By clustering dxgroups with an interval of roughly 500 Euros between the lowest and highest coefficient, a manageable number of 26 DCGs are formed. These clusters are relatively stable and consist of dxgroups that have their payment weights adjusted for multi-qualification. Thus, the primary/secondary distinction is removed from the DCGs and individuals can be assigned multiple times to multiple DCGs. As a result, health spending of individuals with multimorbidity can be more effectively compensated through RE. The model containing these new DCGs will be referred to as Model 2 (M2). To evaluate the impact of this new clustering method on the explanatory power we compare the R^2^ and CPM of M2 with those of M1.

Moreover, to further examine the effects of the new clustering method, we evaluate the impact on proximations of risk selection incentives. More specifically, we calculate the average under/overcompensations for subgroups of the population for the different models applied in this study. To analyze incentives for service-level selection, we calculate the mean financial result per dxgroup under the variousels through (3):

3 $${\text{Mean Financial Result}}\left( {{\text{MFR}}_{{\text{g}}} } \right) = \frac{{\mathop \sum \nolimits_{i = 1}^{{N_{g} }} \left( {\hat{y}_{i} - y_{i} } \right)}}{{N_{g} }}$$

with g representing a particular dxgroup, $\hat{y}_{i}$ the predicted health spending for individual i in group g, $y_{i}$. the actual spending for individual i in group g, and $N_{g}$ the number of individuals in group g. Measure (3) will be calculated for each of the 209 dxgroups and indicates the incentives for insurers to engage in service-level selection. More specifically, a negative value for the MFR for dxgroup g indicates incentives for insurers to select against users of treatments included in dxgroup g (e.g., by not contracting the hospitals/doctors with the best reputation regarding these treatments).

### Step 4: Evaluating the effects of the new DCGs for selective groups

In a final empirical step of this research, the average under/overcompensations are calculated for subgroups defined by chronic illnesses in the GP data. Under/overcompensation for a subgroup of people with a specific chronic illness indicates selection incentives towards that group. Insurers in the Netherlands have several tools to select in favor or against specific groups, e.g., through selective advertisement, group arrangements, supplementary insurance and customer service (Van Kleef et al., 2020a; Van Veen et al., 2015). The GP data covers nearly 700 conditions classified by the ICPC. However, for this particular study only 109 that are labeled ‘chronic’ will be included because the RE system is primarily designed to account for recurring expenditures related to chronic illness. The 109 conditions are selected for this study as they are—according to medical experts—unlikely to be fully recoverable from, and thus relate to future health care spending (Nielen et al., 2019). A complete list of these ICPC conditions is provided in the appendix (Table 2).

Similar to the use of (3) for dxgroups to find the service-level selection incentives, we calculate the mean financial result for each of the 109 chronic conditions in the GP data under the various models. While the mean financial results for the dxgroups specifically indicate incentives for service-level selection through the direct link with treatments, the mean financial results for chronic conditions more generally indicate incentives for group-level selection (Layton et al., 2018a).

## Results

This section presents the results of our analyses. We first report some descriptive statistics of the datasets. Then, the measures of fit are reported for the various models, followed by the effects of the modifications to the baseline model for the dxgroups and ICPC conditions respectively.

### Step 1: Descriptive statistics, rebalancing and the RE model of 2020

Table 1 presents a selection of descriptive statistics for both the total population and the GP data. Despite the notable difference in size, the GP sample differs marginally from the population on the underlying characteristics. The individuals in the GP data are slightly older and have elevated proportions of morbidity adjuster qualifications, contributing to a higher mean spending. After applying the rebalancing process, as discussed in the Data section, the frequencies of risk classes in the GP data are identical to those of the population. Although the rebalancing process substantially reduced the difference in mean spending between the datasets, a difference of roughly 5 Euros remained. In order to offset this leftover difference, we applied a linear correction to the spending for the individuals included in the GP sample.

**Table 1:** Descriptive statistics of the datasets

	Population	Sample (GP data)
Number of individuals	16,873,980	1,308,301
Number of insured years	16,670,015	1,299,534
Mean spending in Euros per year	€2,333	€2,388
Men, 1–17 years	9.8%	9.8%
Men, 18–34 years	10.5%	10.2%
Men, 35–44 years	6.0%	6.0%
Men, 45–54 years	7.6%	7.7%
Men, 55–64 years	6.8%	6.9%
Men, 65 years and older	8.8%	8.9%
Women, 1–17 years	9.4%	9.3%
Women, 18–34 years	10.3%	10.3%
Women, 35–44 years	6.2%	6.2%
Women, 45–54 years	7.6%	7.7%
Women, 55–64 years	6.8%	6.9%
Women, 65 years and older	10.4%	10.3%
Pharmacy-based cost groups	16.9%	17.4%
Primary Diagnosis-based cost groups	9.0%	9.2%
Secondary Diagnosis-based cost groups	3.9%	4.1%
Durable medical equipment cost groups	3.8%	3.8%
Physiotherapy diagnosis groups	1.9%	2.0%
Multiple year high cost groups	6.1%	6.3%
Prior home care spending	2.4%	2.5%
At least one morbidity adjuster	24.9%	25.4%

Spending is presented in euros per person per year. Frequencies are calculated as a percentage of total insured years in the population and (rebalanced) sample, respectively. Individuals aged 0 in 2017 are excluded from both datasets

Moreover, since the GP data is based on diagnoses signaled in 2016 and the population data refers to spending in 2017, individuals aged 0 in 2017 are excluded from the later dataset. Furthermore, as consumers may, for particular reasons, cancel their health insurance at any point in the year, the expenditures on healthcare are annualized and weighted by the fraction of the year the individual was enrolled. Health spending incurred by individuals that were only registered in the first three months, for instance, are therefore quadrupled and included in the analyses with a weight of 0.25.

### Step 2: Revision of dxgroups underlying DCGs

**Fig. 1:**
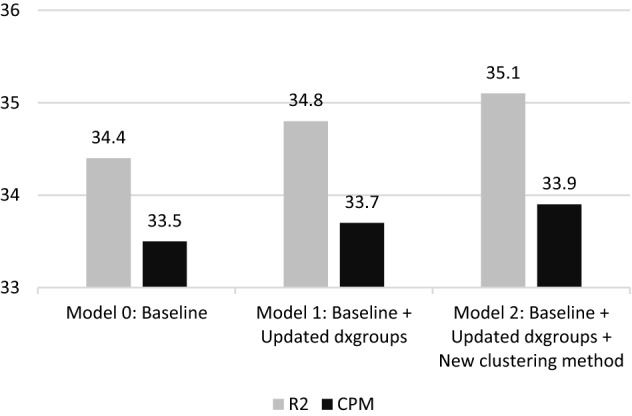
R^2^ and CPM (both × 100) of the three simulated RE models (N = 17 m)

The revision of dxgroups led to the removal of ten dxgroups and the inclusion of nineteen new ones. For the exact reasoning behind these modifications, we refer to the full report for a complete overview of the resulting 209 dxgroups (Van Kleef, 2020b). The effects of this seemingly minor update on the explanatory power of the model are presented in Fig. 1. The R^2^ increases by 0.4 percentage point, indicating that a larger share of variance in health spending is explained by the RE model. Moreover, the update of the dxgroups results in a 0.2 percentage point decrease of absolute differences between actual and predicted health spending, as shown by the increased CPM.

### Step 3: Developing and testing a new method for clustering dxgroups into DCGs

The update of the dxgroups and the new clustering method transformed the 15 primary and 7 secondary DCGs into 26 new separate DCGs that accommodate multi-classification. The latter means that individuals can be classified in multiple DCGs as well as multiple times in the same DCG (i.e., when they fall in multiple dxgroups included in that DCG). Moreover, the total share of the population that qualifies for compensation through DCGs increases from 10.5 to 11.4%. As a result, a larger portion of variance in health spending is explained, reflected by the increase in the measures of explanatory power: the R^2^ and CPM are respectively 0.3 and 0.2 percent point higher for M2 than for M1 (Fig. 1). The R2 and CPM of model M2—with the 209 dxgroups clustered into 26 DCGs—appeared to be nearly identical to those of the same model containing each dxgroup as a separate dummy (results not shown here). This indicates that the new clustering method, while lifting complexity and stability concerns, does not cede explanatory power.

**Fig. 2:**
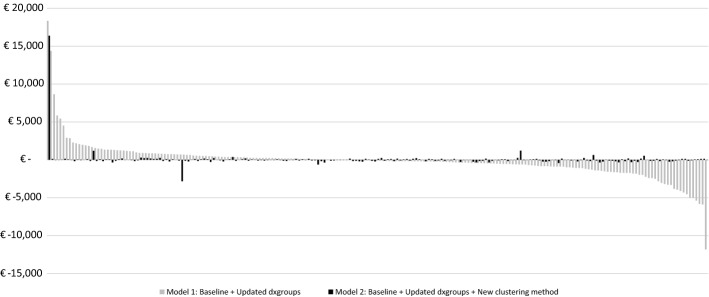
Mean financial result per individual dxgroup (N = 17 m) Mean financial result (i.e., level of under/overcompensation) for the 209 dxgroups, paired for the two models, based on the declining rank under Model 1. Mean result is calculated as the mean predicted spending per person per year minus the mean actual spending per person per year

In Fig. 2, the mean financial results (MFRs) for all 209 dxgroups are projected for M1 and M2. The order of the dxgroups is based on the declining results under M1. Since the dxgroups in the figure are paired for the two models, the results for M2 do not need to follow the trend for M1. In M1 44% of the dxgroups have a mean financial result between €500 and €-500, while in M2 over 97% fits within that range, indicating an improvement in predictive accuracy. Furthermore, of the total number of negative results for both models, 114 and 119 respectively, only 26% is between €0 and €-250 for M1, while 89% of the undercompensations in M2 are in that bracket. By expanding the lower threshold to €-500, those proportions change to 41% and 98%, indicating a noteworthy improvement.

However, a few dxgroups stand out and deviate notably from zero. This is an effect of clustering some small dxgroups with very high coefficients that are not very close to one another. This inherently leads to relatively large under/overcompensations.

The outcomes in Fig. 2 can be summarized by the Weighted Mean Absolute Financial Result (WMAFR) across the 209 dxgroups. This overarching measure can be applied to reflect how capable the model is to predict the actual mean spending for the 209 groups, taking into account the relative size of those groups. Moving from M1 to M2 results in a drop of the WMAFR from €653 to €113, indicating a substantial overall improvement at the level of dxgroups, in line with the findings from Fig. 2.

**Fig. 3:**
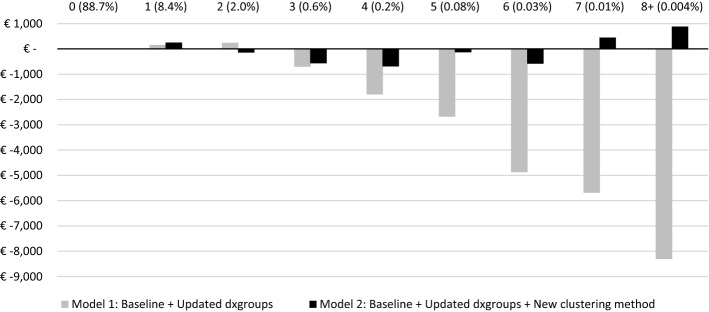
Mean financial result for groups based on the number of dxgroups (N = 17 m) Mean financial result (i.e., level of under/overcompensation) by number of dxgroups in the population sample. Percentages refer to relative frequencies. Mean result is calculated as the mean predicted spending per person per year minus the mean actual spending per person per year

Figure 3 presents the MFR for partitions of the population based on the number of dxgroups that individuals qualify for. The first group (from the left) represents people who are not in any dxgroup (88.7% of the population), followed by groups that are comprised of individuals that are in either 1, 2, 3, 4, 5, 6, 7 or 8 + dxgroups. Under Model 1, the MFRs for the selected groups deviate further from zero for individuals that qualify for more than two dxgroups. For people in more than two dxgroups, Model 2 performs substantially better than the traditional approach of Model 1, indicating that allowing for multi-classification improves the outcomes of RE for people with multiple morbidities.

### Step 4: Evaluating the effects of the new clustering for disease groups

The final methodological step of this research repeats the preceding analyses performed for the 209 individual dxgroups for the 109 ICPC diagnoses extracted from the GP dataset. Thereby, the effects of the new clustering method are evaluated at the level of common chronic conditions, rather than the more specific level of hospital diagnoses. While a complete overview of the results for the 109 individual ICPC groups under both the baseline model and M2 is provided in the appendix (Table 2), the key findings are reported below.

First, we made a partition between individuals that are diagnosed with at least one chronic illness of the ICPC list and those that have no such illness. The former group, the chronically ill individuals, are undercompensated by €80 on average in M0, while those without any diagnosis are overcompensated by €96. Model 2 produces an average result for these two groups of €-79 and €95 respectively, demonstrating a minimal effect of the new DCGs for this general partition of the population.

**Fig. 4:**
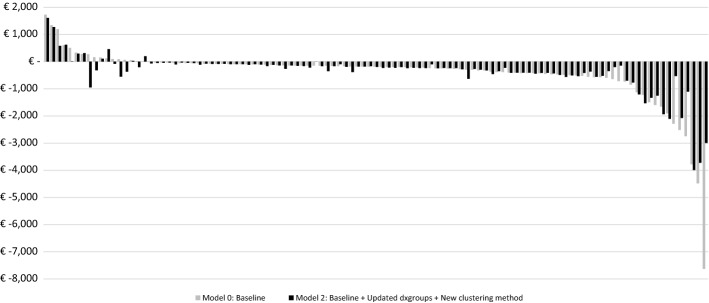
Mean financial result per individual ICPC diagnosis identified in the GP data (N = 1.3 m) Mean financial result (i.e., level of under/overcompensation) for the 109 ICPC groups, paired for the two models, based on the declining rank under Model 0. Mean financial result is calculated as the mean predicted spending per person per year minus the mean actual spending per person per year

Figure 4 displays the MFRs derived for the 109 ICPC diagnoses for both the baseline model and Model 2. The results are paired and ordered based on the (declining) financial results of M0. The differences between the results for the two models are less obvious for these ICPC diagnoses than for the dxgroups shown in Fig. 2. For some of the diagnoses the MFR is closer to 0 for M2 than for M0; for other diagnoses, however, the opposite holds true. This may be explained by the fact that the new clustering method creates new clusters of dxgroups. Assume that some dxgroups were overcompensated before as a result of the inherent heterogeneity problems of the old method and the new DCGs reduce these overcompensations. ICPC diagnoses correlated to these dxgroups are compensated to a lesser extent, producing a different financial result on average.

Nevertheless, while the baseline model produces a statistically significant MFR (*p* < 0.01) that is below zero for 38 diagnoses, model M2 reduces that number to 33. Moreover, moving from M0 to M2 results in an MFR that is statistically significantly different (*p* < 0.05) from the respective MFR produced under the baseline model for five diagnoses. Four (B73, B74, F84 & R84) of these have their undercompensations reduced by roughly 67%, tackling the problematically unprofitable aspect of these specific conditions (see the appendix (Table 2) for the details).

Similar to our earlier calculation for the 209 dxgroups in step 3, the impact of the new clustering method for the 109 ICPC diagnoses can be summarized by the WMAFR: €232 for M0 and €225 for M2, a marginal 3% improvement. The limited effect on the WMAFR can be partly explained by the dissimilar impact on the 109 diagnoses. Additionally, the visible changes between the models at the far right of Fig. 4 are diagnoses with minor frequencies, whereas the hardly changed diagnoses in the center are the most prevalent.

**Fig. 5:**
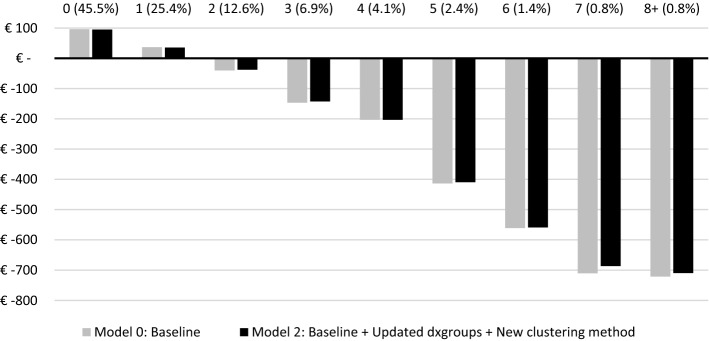
Mean financial result for groups based on the number of chronic conditions identified in the GP data (N = 1.3 m) Mean financial result (i.e., level of under/overcompensation) by number of ICPC diagnoses in the rebalanced GP sample. Percentages refer to relative frequencies. Mean result is calculated as the mean predicted spending per person per year minus the mean actual spending per person per year

Figure 5 presents the MFRs for groups of individuals based on the number of ICPC diagnoses, ranging from 0 up to 8 + . In the figure, a modest difference can be observed between the bars for the subgroups of the two models. All in all, moving from the baseline model to M2 seems to have a minimal effect on the financial result of the selected groups based on the ICPC diagnoses, both in terms of multimorbidity as well as for the overall result for (not) chronically ill consumers.

## Discussion

The findings of this study demonstrate how the Dutch RE model can be improved through a redesign of the DCGs. By applying a new clustering method and allowing for multi-classification, the explanatory power of the RE model at the individual level is increased. More specifically, we find an increase of the R^2^ (× 100) from 34.4 to 35.1. Given the level of sophistication of the Dutch model (with multiple risk adjusters that collectively incorporate over 200 indicators) this 0.7 increase can be considered a meaningful improvement since major gains on this measure are rarely made in advanced models (Layton et al., 2018a). At the level of dxgroups the new clustering method results in substantial reductions of under/overcompensation, implying that the method better accounts for spending heterogeneity among dxgroups. For subgroups defined by ICPC diagnoses from GPs, improvements are relatively minor. The main explanation is that the new design of the DCGs does not extend the group of people qualifying for compensation through a DCG but ‘only’ improves compensation among those that already did qualify. In other words: the new design moves funds *within* the group of people qualifying for a DCG but does not increase funds for this group as a whole. Although under/overcompensations reduce for some ICPC diagnoses, they increase for others, implying that the overall effect for these disease groups is limited.

All in all, it can be stated that the new approach for compiling DCGs is a successful route to improve the predictive accuracy of the Dutch model, both at the level of individuals and at the level of subgroups. In general, these improvements mean that selection incentives for insurers will be reduced since the under/overcompensations shrink (Van Vliet et al., 2015). With respect to the two sets of subgroups identified in this research (based on either the dxgroups or the ICPC diagnoses) we distinguish incentives for service-level selection and group-level selection (Layton et al, 2018a). With respect to the broader, group-level incentives derived from the ICPC diagnoses, the new DCGs produce marginal differences from the present (2021) model to stimuli for risk selection regarding disease groups. In contrast, incentives for service-level selection are more effectively addressed by our method. Since contracting of care is performed at the treatment level, the financial results for the dxgroups serve as a fitting proxy for selection incentives. By rendering the majority of dxgroups equally attractive in financial terms, as shown in Fig. 2, our new DCG approach thus substantially reduces incentives for service-level selection in Dutch basic health insurance.

Despite the advancements made in reducing under/overcompensations for treatment groups, the Dutch RE system persistently undercompensates the overall group of chronically ill individuals, indicating that risk selection incentives towards this ambiguous group remain. A potential explanation could be that while—according to the GP patient records—55% of Dutch individuals have a chronic condition, only 25% of the population qualifies for at least one morbidity adjuster. A substantial share of the chronically ill are not flagged by any morbidity indicator, resulting in inadequate financial compensation. The undercompensation of diabetics (€-194 under M2), for instance, may result from the disparity in its prevalence between the GP data (6.1%) and its presence in the morbidity adjusters in the RE model (less than 5%). The resulting incentives undermine the goals of efficiency and fairness in the Dutch health insurance market, accentuating the need for further improvements of the RE model. As long as groups of consumers in need of specific types of care remain predictably unprofitable, insurers face a disincentive to improve the quality of these specific types of care.

Additional research is required to detect the individuals that are chronically ill but are not found by the current RE model. While our study refines one morbidity adjuster and addresses the heterogeneity within, it is inadequate to eliminate undercompensations for all chronically ill individuals. Potentially, new risk adjusters may yield explanatory value by including more extensive information on health or illness. Despite the fact that the GP data used in this study only covers a portion of the entire population, its usefulness should not be disregarded. Through the gatekeeping role of GPs in the Dutch health system, they possess information on the health status of individuals that may, for any particular reason, not use secondary care. Since the morbidity adjusters in the RE system are mostly based on information through hospital treatments and drug prescriptions, this could result in the mismatch between a signaled illness in the GP data and no compensation through RE. The GP data could be used for the RE model when made representative for the entire population or efforts could be made to acquire this particular information for all individuals in Dutch basic health insurance. By doing so, the model can explicitly compensate chronic illnesses, as indicated by the GP, rather than implicitly through hospital treatments or drug prescriptions.

Alternatively, instead of using the ICPC information for new morbidity adjusters, another promising strategy to reduce the incentives for risk selection is by applying new estimation techniques. Constrained regression, for instance, can be used to enhance the financial result for specific subgroups, such as the ICPC diagnoses (Van Kleef et al., 2020a). The model could then be calibrated to ensure a mean financial result for diabetics, or any other group, of €0 or even a profit. By using the GP information in such ways, the selection incentives towards these groups can be diminished. Another alternative may lie in shifting from the prospective nature of the Dutch RE model to a concurrent model, not based on historical information but rather based on data from the current year. Information from the current year may be notably more accurately reflect the health status of an individual than historical data. This particular transition does, however, result in a larger degree of endogeneity between healthcare expenditures incurred and compensation received. These options are interesting directions for further research.

Nevertheless, by allowing for multi-classification, the Dutch DCGs become more in line with diagnostic classifications used in RE models in the U.S. and Germany (i.e. the Hierarchical Condition Categories and the Hierarchical Morbidity Groups, respectively). One important difference, however, remains: while the U.S. and Germany classifications rely on direct ICD-codes, the Dutch model uses context-specific ‘diagnosis treatment combinations’ (DTCs). Unfortunately, these DTCs do not allow for building disease hierarchies that take disease severity into account. It is uncertain, however, if and when ICD-codes become directly available for the purpose of RE in the Netherlands; once they do, it should be possible to further refine the Dutch DCGs. Still, the new DCG approach of this study is notably effective in reducing the incentives for service-level selection, contributing to the functioning of the health insurance market. Although this study focuses on the relatively specific Dutch context, the findings and the considerations for further improvement may be valuable to regulators or health insurance systems elsewhere seeking to apply RE to counteract risk selection incentives. The overall takeaway from our analyses is that smart design of morbidity adjusters can help mitigate selection incentives.

## Data Availability

The used data is strictly confidential and requires a signed agreement of permission by the Dutch Ministry of Health, Welfare and Sports and the Central Bureau for Statistics. *Therefore, it would fit to not further specify the data availability for the publication.*

## Appendix

See Table 2

**Table 2:** Mean financial result for the 109 chronic conditions identified in the GP data (N = 1.3 m)

Code	Chronic condition	Relative frequency	Mean spending per person per year (€)	Mean financial result per person for Model 0 (€)	Mean financial result per person for Model 2 (€)
A28	Limited function/disability NOS	0.1	7,199	− 485	− 561
A79	Malignancy NOS	0.1	17,211	**− 4,483	**− 3,720
A90	Congenital anomaly OS/multiple	0.2	5,262	− 177	− 387
B28	Limited function/disability blood and/or blood forming organs	0.0	7,608	− 1,498	− 1,329
B72	Hodgkin's disease/lymphoma	0.2	9,740	89	− 556
B73	Leukemia	0.1	15,917	**− 2,285	− 530
B74	Malignant neoplasm blood other	0.1	24,849	**− 7,627	**− 2,991
B78	Hereditary haemolytic anaemia	0.2	3,051	− 145	− 163
B79	Congen. anomaly blood/lymph other	0.0	8,145	**− 1,597	*− 1,250
B83	Purpura/coagulation defect	0.6	6,311	− 255	− 241
B90	HIV− infection/aids	0.1	14,995	*− 573	*− 522
D28	Limited function/disability	0.0	9,136	− 153	− 349
D74	Malignant neoplasm stomach	0.1	9,212	* 1,194	582
D75	Malignant neoplasm colon/rectum	0.7	9,534	− 137	− 268
D76	Malignant neoplasm pancreas	0.0	16,621	*− 2,516	− 2,076
D77	Malig. neoplasm digest. other/NOS	0.2	11,275	**− 1,226	**− 1,531
D81	Congen. anomaly digestive system	0.3	2,120	*− 211	*− 228
D92	Diverticular disease	1.7	6,182	**− 192	**− 232
D94	Chronic enteritis/ulcerative colitis	0.8	6,761	**− 401	**− 413
D97	Liver disease NOS	0.8	5,868	**− 571	**− 558
F28	Limited function/disability eye	0.2	4,563	− 33	− 43
F81	Congenital anomaly eye other	0.2	2,428	− 103	− 108
F83	Retinopathy	0.7	9,437	**− 559	*− 366
F84	Macular degeneration	0.6	9,036	**− 640	− 202
F91	Refractive error	3.7	2,540	− 54	− 54
F93	Glaucoma	1.5	5,255	− 33	− 49
F94	Blindness	0.2	6,020	− 325	− 324
H28	Limited function/disability ear	0.1	4,454	329	293
H80	Congenital anomaly of ear	0.1	2,782	− 453	*− 413
H83	Otosclerosis	0.1	3,374	− 199	− 241
H84	Presbyacusis	1.7	7,184	− 79	− 84
H85	Acoustic trauma	0.3	4,212	− 194	− 218
H86	Deafness	2.0	5,012	**− 216	**− 238
K28	Limited function/disability cardiovascular	0.1	5,435	− 422	− 405
K73	Congenital anomaly cardiovascular	0.4	3,341	156	108
K74	Ischaemic heart disease w. angina	2.3	7,846	**− 249	**− 231
K76	Acute myocardial infarction	1.1	7,934	**− 382	**− 351
K77	Heart failure	1.1	14,100	**− 849	**− 768
K82	Pulmonary heart disease	0.0	17,436	− 1,126	− 1,205
K86	Hypertension uncomplicated	14.0	5,116	**− 182	**− 183
K87	Hypertension complicated	1.8	7,982	**− 468	**− 436
K90	Stroke/cerebrovascular accident	1.7	8,838	**− 366	**− 459
K91	Atherosclerosis [ex. K76,K90]	1.2	6,467	**− 301	**− 269
K92	Cardiovascular disease other	2.1	7,426	**− 731	**− 700
L28	Limited function/disability musculoskeletal	0.3	7,428	**− 506	**− 536
L82	Congenital anomaly musculoskeletal	1.0	2,226	− 60	*− 110
L84	Back syndrome w/o radiating pain	1.5	6,148	**− 195	**− 229
L85	Acquired deformity of spine	0.8	3,519	*− 170	**− 192
L88	Rheumatoid/seropositive arthritis	1.3	7,411	− 148	**− 220
L89	Osteoarthrosis of hip	2.3	7,196	**− 496	**− 509
L90	Osteoarthrosis of knee	3.3	6,783	**− 425	**− 445
L91	Osteoarthrosis other	2.9	5,507	**− 196	**− 199
L95	Osteoporosis	2.6	6,978	**− 219	**− 235
L98	Acquired deformity of limb	3.9	6,186	**− 130	**− 138
N28	Limited function/disability neurological system	0.0	5,939	98	− 81
N70	Poliomyelitis	0.0	6,602	− 9	198
N74	Malignant neoplasm nervous system	0.1	10,145	164	− 321
N85	Congenital anomaly neurological	0.1	9,068	273	*− 949
N86	Multiple sclerosis	0.2	11,702	− 152	5
N87	Parkinsonism	0.3	12,234	− 263	− 235
N88	Epilepsy	1.0	5,508	− 52	− 34
P28	Limited function/disability psychological	0.1	3,940	− 180	− 184
P70	Dementia	0.6	7,423	** 1,341	** 1,274
P72	Schizophrenia	0.3	3,606	* 284	** 313
P80	Personality disorder	1.0	3,375	**− 161	**− 167
P85	Mental retardation	0.5	3,514	− 47	− 103
R28	Limited function/disability respiratory system	0.1	8,682	− 239	− 102
R84	Malignant neoplasm bronchus/lung	0.2	17,065	**− 2,747	**− 1,104
R85	Malignant neoplasm respiratory other	0.1	11,430	**− 1,656	**− 1.,937
R89	Congenital anomaly respiratory	0.0	7,828	− 1,902	− 2,104
R91	Chronic bronchitis/bronchiectasis	0.8	5,822	*− 247	*− 245
R95	Chronic obstructive pulmonary disease	2.6	8,339	**− 422	**− 406
R96	Asthma	8.9	2,983	**− 77	**− 87
S28	Limited function/disability skin	0.0	4,461	− 95	− 117
S77	Malignant neoplasm of skin	3.3	5,638	**− 169	− 97
S81	Haemangioma/lymphangioma	0.8	2,592	− 80	− 80
S83	Congenital skin anomaly other	0.3	2,648	− 101	− 97
S87	Dermatitis/atopic eczema	9.2	2,123	*− 36	*− 35
S91	Psoriasis	2.4	4,274	**− 187	**− 169
T28	Limited function/disability endocrine system	0.0	11,103	− 3.,776	− 3,991
T71	Malignant neoplasm thyroid	0.1	5,739	121	462
T78	Thyroglossal duct/cyst	0.1	3,077	− 122	− 118
T80	Congenital anom. endocrine/metab	0.1	7,177	− 473	− 492
T81	Goitre [ex. T85,T86]	0.5	4,565	− 95	− 97
T86	Hypothyroidism/myxoedema	2.6	4,746	*− 92	*− 95
T90	Diabetes	6.1	7,221	**− 188	**− 194
T92	Gout	2.5	6,413	**− 405	**− 407
T93	Endocrine/metab./nutrit. dis. other	7.8	4,550	**− 141	**− 140
U28	Limited function/disability urinary	0.1	16,265	− 294	− 634
U75	Malignant neoplasm of kidney	0.1	11,545	*− 721	− 148
U76	Malignant neoplasm of bladder	0.3	9,757	68	− 370
U77	Malignant neoplasm urinary other	0.0	9,692	− 107	− 164
U85	Congenital anomaly urinary tract	0.2	4,310	− 153	− 168
U88	Glomerulonephritis/nephrosis	0.1	7,638	− 382	− 228
W28	Limited function/disability due to pregnancy	0.1	2,101	52	27
W72	Malignant neoplasm related to pregnancy	0.0	615	**1,728	**1,610
W76	Congenital anomaly complicate pregnancy	0.0	2,344	− 276	− 281
X28	Limited function/disability female genital	0.0	2,563	604	623
X75	Malignant neoplasm cervix	0.2	4,555	**− 427	*− 416
X76	Malignant neoplasm breast female	1.3	6,815	19	*− 208
X77	Malignant neoplasm female genital other	0.2	7,411	*− 524	− 413
X83	Congenital anomaly female genital	0.1	2,426	− 324	− 297
X88	Fibrocystic disease breast	1.4	2,712	− 87	*− 97
Y28	Limited function/disability male genital	0.1	4,713	− 13	− 67
Y77	Malignant neoplasm prostate	0.6	8,819	**− 598	*− 347
Y78	Malign. neoplasm male genital other	0.1	3,980	505	− 9
Y82	Hypospadias	0.1	1,849	− 52	− 45
Y84	Congenital genital anomaly male other	0.1	1,632	− 77	− 77
Z28	Limited function/disability social	0.3	3,820	− 144	− 157
Weighted Mean Absolute Deviation (WMAD) (€)				231.6	224.8

Results are based on the rebalanced sample (N = 1.3 m). A positive (negative) financial result indicates an average profit (loss) to the insurer. ** = statistically significantly different from zero with *p* < 0.01. * = statistically significantly different from zero with 0.01 < *p* < 0.05. The WMAD is derived by the summation of all absolute average residuals multiplied by their respective number of insured years, subsequently divided by the summation of the insured years for the 109 diagnoses

## Footnotes

1 In practice, the Dutch RE model consists of three components: one model for somatic care, one for mental care and one for out-of-pocket spending due to a mandatory deductible. See Van Kleef et al. (2018) for more detail. The DCGs discussed in this paper are part of the model for somatic care.

## Code availability

Upon request.
